# Resistance Genes and Virulence Factor Genes in Coagulase-Negative and Positive Staphylococci of the *Staphylococcus intermedius* Group (SIG) Isolated from the Dog Skin

**DOI:** 10.3390/microorganisms13040735

**Published:** 2025-03-25

**Authors:** Simona Hisirová, Jana Koščová, Ján Király, Vanda Hajdučková, Patrícia Hudecová, Stanislav Lauko, Gabriela Gregová, Nikola Dančová, Júlia Koreneková, Viera Lovayová

**Affiliations:** 1 Department of Microbiology and Immunology, The University of Veterinary Medicine and Pharmacy in Košice, 041 81 Kosice, Slovakia; simona.hisirova@student.uvlf.sk (S.H.); jana.koscova@uvlf.sk (J.K.); jan.kiraly@uvlf.sk (J.K.); vanda.hajduckova@uvlf.sk (V.H.); patricia.hudecova@student.uvlf.sk (P.H.); stanislav.lauko@uvlf.sk (S.L.); 2 Department of Public Veterinary Medicine and Animal Welfare, The University of Veterinary Medicine and Pharmacy in Košice, 041 81 Kosice, Slovakia; gabriela.gregova@uvlf.sk (G.G.); nikola.dancova@uvlf.sk (N.D.); 3 Department of Nutrition and Food Quality Assessment, Institute of Food Science and Nutrition, Faculty of Chemical and Food Technology, Slovak University of Technology, Radlinského 9, 812 37 Bratislava, Slovakia; julia.korenekova@stuba.sk; 4 Department of Medical and Clinical Microbiology, Faculty of Medicine, Pavol Jozef Šafárik University in Košice, Trieda SNP 1, 040 11 Kosice, Slovakia

**Keywords:** *Staphylococcus intermedius* group, coagulase-negative staphylococci, shelter dogs, seasonality, biofilm production, antibiotic resistance, PCR, MALDI-TOF MS, MIC, *mecA*

## Abstract

Staphylococci are common pathogens in dogs, causing a variety of dermatological problems. This study aimed to characterize the prevalence, antibiotic resistance, and biofilm-forming potential of *Staphylococcus* species isolated from the skin of shelter dogs. Overall, 108 samples were collected from the hairless skin areas of dogs in a shelter over one year. Isolates were cultured using standard microbiological methods and identified through biochemical testing, MALDI-TOF MS, and multiplex PCR. A total of 67 *Staphylococcus* isolates were identified, with *S. pseudintermedius* being the most prevalent. Antibiotic susceptibility was assessed using disk diffusion and MIC methods, revealing high resistance to ampicillin, erythromycin, and tetracycline. Notably, 12 multidrug-resistant SIG (*S. intermedius* group; *S. pseudintermedius*) and 4 CoNS strains (coagulase-negative staphylococci; *S. equorum*) were identified. Biofilm production was evaluated using a crystal violet assay, showing variable biofilm-forming capabilities among isolates and PCR, to confirm genes associated with biofilm formation. These findings highlight the presence of multidrug-resistant *Staphylococcus* species in shelter dogs, emphasizing the need for careful monitoring and antibiotic stewardship to manage potential risks to both animal and human health.

## 1. Introduction

Staphylococci are among the most important pathogens for humans and animals [1]. They are the causative agents of many diseases, especially pyogenic skin infections, soft tissue infections, respiratory infections, and sepsis [2]. The skin is constantly in contact with the environment and other individuals, so it represents a suitable place for the growth and interspecific spread of bacteria and is an ideal habitat, with a prevalence of resistant strains [3]. For a long time, attention has been paid to coagulase-positive (CoPS) [4] *S. aureus*. Currently, scientific studies focus more on other CoPS, especially SIG (*Staphylococcus intermedius* group), which includes *S. pseudintermedius*, *S. intermedius*, *S. delphini*, *S. cornubiensis*, and *S. ursi* [5], and opportunistic pathogenic coagulase-negative staphylococci (CoNS) [6,7]. Recent studies have proven that humans and companion animals are often reservoirs and can spread strains of staphylococci to each other [8,9]. Shelter dogs frequently experience elevated stress levels, inadequate hygiene, overcrowding, and frequent interactions with other animals, all of which can lead to colonization by harmful microorganisms, such as staphylococci, and increased susceptibility to infections. Furthermore, the restricted availability of veterinary care in shelters may lead to untreated infections and the possible dissemination of resistant strains in these settings [10]. One potentially risky bacterium belonging to SIG is *S. pseudintermedius*, which occurs in dogs as a natural skin commensal [11,12]. From the perspective of the risk of transmission from dogs to humans, CoNS are also significant, especially novobiocin-sensitive staphylococci, e.g., *S. simulans*, *S. epidermidis*, *S. xylosus*, and *S. condimenti* and novobiocin-resistant staphylococci, such as *S. saprophyticus*, *S. equorum*, *S. warneri*, *S. sciuri*, *S. vitulinus*, etc. [13]. Resistance to antibacterial agents is becoming a problem in selecting an appropriate therapeutic agent for the treatment of infections caused by resistant staphylococci to a wide range of antibiotics from different pharmacotherapeutic groups, including reserve antibiotics [14].

Due to resistance to antibacterial agents encoded by plasmid DNA or other mobile gene elements, the horizontal transfer of genetic information encoding resistance can occur in bacterial species that were initially sensitive to these antibiotics [14]. For example, methicillin resistance is caused by the expression of the *mecA* or homologous *mecC* genes, which are part of the staphylococcal cassette chromosome *mec* (*SCCmec*) [15,16]. The product of these genes is a modified low-affinity penicillin-binding protein (PBP 2A), which functions as a transpeptidase [17]. Another important group of enzymes is the extended spectrum beta-lactamases (ESBLs) [18,19].

Many staphylococcal infections are caused by strains capable of producing biofilms. A biofilm is a microbial colony of cells attached to a biotic or abiotic surface protected by an extracellular polymeric substance (EPS; glycocalyx) [20,21]. Biofilm formation is a process characterized by several phases [22]. In the first phase, bacterial adhesion to the host tissue occurs, which is responsible for bacterial cell wall proteins MSCRAMMs (microbial surface components recognizing adhesive matrix molecules), belonging to a large group of CWAPs (cell wall-anchored proteins), which interact with host extracellular proteins [23,24,25,26]. A common feature of MSCRAMM is the amino acid motif LPXTG (leucine-proline-any amino acid-threonine-glycine), recognizing and cleaving by the enzyme sortase A (encoded by the *srtA* gene), which plays a role in the attachment of CWAPs to the peptidoglycan [27,28]. As a key step in infection development, important adhesins are fibronectin-binding proteins A and B (FnbA, FnbB; encoded by *fnbA* and *fnbB* genes) [29]. The adhesion process is also attended by the clumping factors ClfA (ligands are fibrinogen and complement factor I) and ClfB (ligands are cytokeratin 8 and 10) encoded by *clfA* and *clfB* genes [30,31]. After the adhesion of bacterial cells, an accumulation phase mediated by PIA (polysaccharide intercellular adhesin) follows, whose synthesis encodes the chromosomal intercellular adhesion (*ica*) locus, which consists of the structural genes of the *icaADBC* operon and the regulatory gene *icaR*. The most important parts of the *icaADBC* operon are products of the *icaA* and *icaD* genes, which play a role in biofilm formation due to their mutually potentiated enzymatic activity [32,33]. During the final phase of biofilm dispersal, cells detach and structure the biofilm by remodeling [21]. The entire process controls the accessory gene (*agr*) regulatory operon, which encodes the Agr as a part of the *quorum sensing* system (QS)—a bacterial communication process between cells. Bacteria perceive changes in cell population density through AIP (auto-inducting peptide), which activates Agr components [34,35]. Agr consists of two transcriptional units: the P2 promoter controls the expression of the transcriptional activator of the *agr* operon (*agrBDCA*), the product of which is RNA II, and the P3 promoter controls the production of RNA III, a regulator of the expression of genes regulated by the Agr system [36].

This study aimed to monitor the prevalence of multidrug-resistant SIG and CoNS in dogs (important companion animals). This included monitoring biofilm formation as a critical virulence factor associated with antibiotic resistance, monitoring antibiotic resistance, and determining genes associated with the aforesaid virulence factor and antibiotic resistance. The main goal of this study was to generate and provide new insights into the phenotypic and genotypic profiles of antibiotic resistance and to assess biofilm formation.

## 2. Materials and Methods

### 2.1. References Strains

*S. aureus* CCM 4223 isolated from the wound was used as a reference for the detection of 16S rRNA, *eap*, and *nuc* genes. The control strains for genes associated with biofilm formation *icaA*/*icaB*/*icaC*, *agrA*/*srtA*/*icaD*, *fnbA*/*fnbB*, and *clfA*/*clfB* were used. *S. aureus* CCM 4223 was isolated from the wound. For the phenotypic detection of the ability to form biofilm, *S. aureus* CCM 4223 was used as a biofilm-forming reference, which isolated from the wound strain, and *S. epidermidis* CCM 4418 was used as a non-biofilm-forming reference strain. *S. aureus* CCM 4750 was the control for detecting the *mecA* gene, which was isolated in the USA, Kansas from a clinical sample. The reference strain for detecting the presence of the *mecC* gene was *S. edaphicus* CCM 8731, which was isolated from sandy soil in Antarctica. All strains were obtained from the Czech Collection of Microorganisms (Brno, Czech Republic) [37,38,39].

### 2.2. Sampling, Culture, and Identification

Samples (*n* = 108) were obtained from hairless skin areas of shelter-housed dogs (groin and wound areas) without pyogenic infection. They were collected throughout the year of 2023 from spring to autumn. Isolates were processed and cultured using traditional microbiological methods, including primary cultivation on blood agar, followed by selective nutrient media MSA (mannitol salt agar; HiMedia Laboratories, Mumbai, India) and BPA (Baird-Parker agar; HiMedia Laboratories, Mumbai, India). Cultivation occurred at 37 °C for 24 h in an incubator. The isolates were then Gram-stained and subjected to phenotypic identification using biochemical tests (including catalase, coagulase, DNase, lecithinase, and fermentation of mannitol and maltose, along with viability in 7.5% NaCl medium and tellurite reduction), along with the STAPHY test 24 biochemical series (Erba Lachema, Brno, Czech Republic). Confirmation of identification involved MALDI-TOF MS analysis of isolates grown on MSA and molecular-level verification via multiplex PCR targeting the 16S rRNA sequence (141 bp) specific to the genus *Staphylococcus*, while *S. aureus* was identified through amplification of the species-specific genes *eap* and *nuc*. Primers amplifying *S. aureus*-specific gene segments ensured that any misidentification by other methods would be detected. Identified strains were stored in microbank cryovials (Pro-Lab, Mississauga, ON, Canada) at −80 °C.

### 2.3. MALDI-TOF MS

Definitive species confirmation was performed using MALDI-TOF MS (matrix-assisted laser desorption ionization–time of flight mass spectrometry; Bruker, Karlsruhe, Germany). Protein isolation and plate preparation were performed following the standard protocol [35]. The steel plate was inserted into an AutoFlex I TOF-TOF instrument (Bruker Daltonics Inc., Billerica, MA, USA). Spectra were analyzed using MALDI BioTyper software (v 2.0, BioTyper Library v 3.0; Bruker Daltonics sro, Brno, Czech Republic) [40].

### 2.4. DNA Extraction

Genomic DNA was extracted from overnight cultures of isolates grown in mBHI (modified brain heart infusion; HiMedia Laboratories, Mumbai, India) containing 1.0% glucose and 2.0% NaCl using the High Pure PCR DNA Extraction Kit (Roche Molecular Systems, Inc., Pleasanton, CA, USA). DNA concentration and purity were determined spectrophotometrically using an ND-8000 system (Thermo Fisher Scientific, Waltham, MA, USA).

### 2.5. Gene Detection Using PCR and Gel Electrophoresis

To confirm genus identification and detect antibiotic resistance and virulence factor genes involved in biofilm formation, simplex and multiplex PCR (mPCR) methods were employed. Primers targeted sequences of the 16S rRNA gene (species identification), *eap* and *nuc* (*S. aureus*-specific genes), *mecA* and *mecC* (genes encoding beta-lactam antibiotic resistance), *bap*, *icaABCD*, *clfA*, *clfB*, *fnbA*, *fnbB* (biofilm formation genes), *srtA* (encoding sortase A, which cleaves surface proteins with the LPXTG motif), and *agrA* (a regulatory gene involved in quorum sensing). Single-primer PCR was performed for *bap*, *mecA*, and *mecC*, while multiplex PCR targeted the following gene sets: 16S rRNA/*eap*/*nuc*, *icaA*/*icaB*/*icaC*, *agrA*/*srtA*/*icaD*, *fnbA*/*fnbB*, and *clfA*/*clfB*. PCR conditions and primer sequences were described by Király et al. [31]. PCR amplification was conducted using a Mastercycler^®^ nexus X2 thermal cycler (Eppendorf, Hamburg, Germany). Electrophoretic separation of nucleic acids was performed on a Wide Mini-Sub^®^ GT Cell electrophoresis system (Bio-Rad, Hercules, CA, USA) using a 2% agarose gel stained with the non-toxic fluorescent dye GoodView™ Nucleic Acid Stain (Amplia, SR, Bratislava, Slovakia). PCR products were visualized under UV light using a UV-Reader Quantum system (Vilber Lourmat, Collégien, France) and analyzed with the VisionCapt digital imaging system (Vilber Lourmat, Collégien, France).

### 2.6. Disk Diffusion Method

The Kirby–Bauer disk diffusion method was chosen for the initial determination of antibiotic susceptibility. Antibiotics, impregnated at appropriate concentrations on 6 mm paper disks, were applied to the surface of Mueller–Hinton agar (MHA; HiMedia Laboratories, Mumbai, India) inoculated with a 24 h bacterial suspension (0.5 McFarland standard) using a sterile needle. After 24 h of incubation at 37 °C, the inhibition zone diameter was measured. The classification of isolates according to inhibition zone diameters followed the guidelines of EUCAST 2024 (version 14.0) [41] and CLSI (document M100-Ed33) [42], categorizing them as susceptible (S), intermediate (I), or resistant (R). The antibiotics tested included amikacin (AK 25 μg), amoxicillin/clavulanate (AMC 30 μg), cephalexin (CN 30 μg), cefovecin (CVN 30 μg), doxycycline (DOX 30 μg), enrofloxacin (ENR 5 μg), clindamycin (CLN 2 μg), and co-trimoxazole (COT 25 μg) (Oxoid, Hampshire, UK).

### 2.7. MIC Determination (Miditech System)

For isolates that exhibited qualitative resistance, antibiotic susceptibility was further assessed quantitatively using the minimum inhibitory concentration (MIC) test for higher precision. MIC values were determined by a colorimetric microdilution method with automated reading via the Miditech system (Bratislava, Slovakia) [43]. The antibiotics tested in the MIC assay included ampicillin (AMP), ampicillin + sulbactam (SAM), piperacillin + tazobactam (TZP), oxacillin (OXA), cefoxitin (FOX), gentamicin (GEN), ciprofloxacin (CIP), moxifloxacin (MFX), erythromycin (ERY), clindamycin (CLI), linezolid (LNZ), rifampicin (RIF), vancomycin (VAN), teicoplanin (TEC), tetracycline (TET), tigecycline (TGC), chloramphenicol (CHL), trimethoprim (TMP), trimethoprim + sulfonamide (COT), and nitrofurantoin (NIT). The Miditech software (Bel-MIDITECH s.r.o., Bratislava, Slovakia; cat. n. 002002) also predicted resistance mechanisms and resistance percentages for each antibiotic. Based on MIC breakpoints following the guidelines of EUCAST 2024 (version 14.0) [41], the system classified isolates as susceptible or resistant and co-trimoxazole (COT) as susceptible, intermediate, or resistant.

### 2.8. Biofilm Activity Assay

Biofilm activity was assessed in a 96-well microtiter plate using a modified colorimetric method based on crystal violet staining, as described by O’Toole et al. [44]. *Staphylococcus* spp. isolates (*n* = 67) were cultivated overnight on blood agar at 37 °C. A bacterial suspension (1 McFarland standard) was prepared from the overnight culture. Then, 100 µL of the bacterial suspension and 100 µL of modified brain heart infusion (mBHI; HiMedia Laboratories, Mumbai, India) were added to each well. *S. aureus* CCM 4223 and *S. epidermidis* CCM 4418 served as reference strains [37,38]. MBHI alone was used as a negative (purity) control. The plates were incubated for 24 h at 37 °C. After incubation, the medium was discarded, and the wells were washed four times with distilled water. Biofilms were then stained with 200 µL of 0.1% crystal violet solution (Merck, Darmstadt, Germany) and incubated at room temperature for 30 min. After incubation, 200 µL of 30% glacial acetic acid was added to each well. The optical density (OD) was measured at 550 nm using a SYNERGY READER 4 (BioTek, Merck, Germany) from three repetitions of a single tested strain, and the diameters were calculated.

### 2.9. Statistical Evaluation of Biofilm Activity

The ability to form biofilms was statistically evaluated against the negative control (*S. epidermidis* CCM 4418) using the method described by Stepanović et al. [45]:

ODc = average OD SE4418 + 3 × SD SE4418(1)

where ODc is the cut-off optical density, SD is the standard deviation, and SE4418 is *S. epidermidis* CCM 4418.

The biofilm-forming ability of the tested strain was determined using the following formula:

OD = average OD of the strain − ODc(2)

where OD is the optical density and ODc is the cut-off optical density.

Statistical analysis was performed using GraphPad Prism 6.01 (GraphPad Inc., San Diego, CA, USA). One-way analysis of variance (ANOVA) followed by Tukey’s test was used to assess significance at *p* < 0.001.

## 3. Results

### 3.1. Isolate Identification

**Figure 1:**
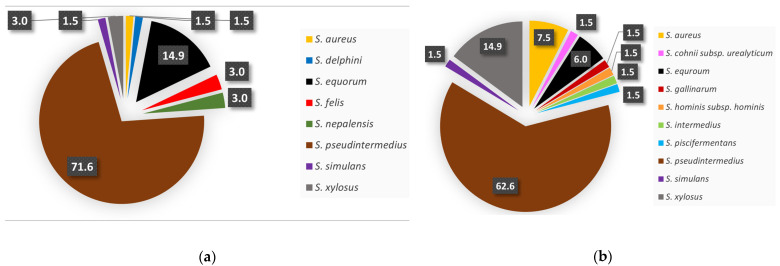
Representation of *Staphylococcus* species isolated from the skin of shelter dogs according to MALDI TOF (**a**) and STAPHY 24 test (**b**).

The most abundant coagulase-positive *Staphylococcus* (CoPS) species among the isolates was *S. pseudintermedius* (62.7%). Other identified CoPS included *S. aureus* subsp. *aureus* (7.5%) and *S. intermedius* (1.5%). The remaining isolates were classified as coagulase-negative *Staphylococcus* (CoNS), specifically *S. xylosus* (14.9%), *S. equorum* (6%), and *S. cohnii* subsp. *urealyticum*, *S. gallinarum*, *S. hominis* subsp. *hominis*, *S. piscifermentans*, and *S. simulans* (each 1.5%) (STAPHY test 24). According to MALDI-TOF MS, *S. pseudintermedius* was again the most abundant CoPS (71.6%), while *S. aureus* subsp. *aureus* (1.5%) and *S. delphini* (1.5%) were also identified. The remaining isolates were classified as CoNS, specifically *S. equorum* (14.9%), *S. xylosus*, *S. felis*, *S. nepalensis* (each 3%), and *S. simulans* (1.5%) (Figure 1). The *S. aureus* isolates identified by the STAPHY test 24 were subjected to confirmatory PCR with the result that only one out of five *S. aureus* isolates was confirmed. As a control strain, we used *S. aureus* CCM 4223.

**Figure 2:**
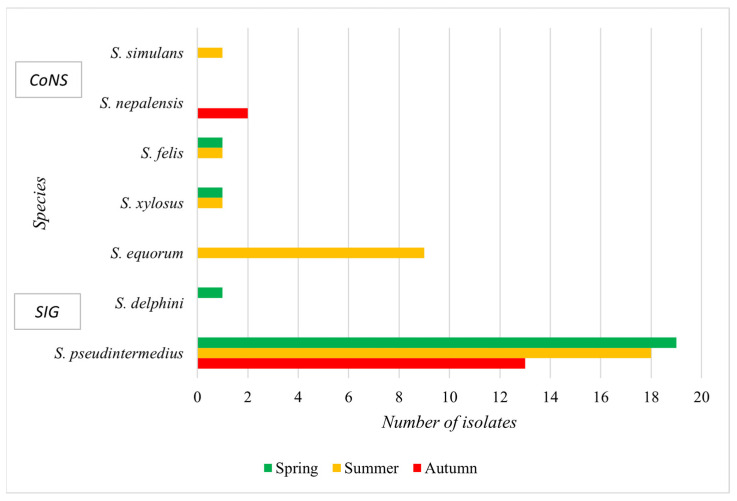
Representation of SIG (*S. delphini*, *S. pseudintermedius*) and CoNS (*S. simulans*, *S. nepalensis*, *S. xylosus*, *S. equorum*) in isolates from shelter dog skin by season. Spring = March–May, summer = June–August, autumn = September–November.

In the samples collected from individual periods, the highest number of *Staphylococcus* spp. isolates were found in the summer months. The prevalence of CoPS was slightly higher in spring (38%) than in summer (36%), while its occurrence was lower in autumn (26.5%). *S. delphini* was isolated only in spring. The prevalence of CoNS was highest in summer, with all isolates of *S. equorum* and *S. simulans* detected during this period, along with 50% of *S. felis* and *S. xylosus* isolates. *S. nepalensis* was found exclusively in autumn (Figure 2).

### 3.2. Determination of Susceptibility to Selected Antibiotics

Among the 67 *Staphylococcus* spp. isolates, 32 clinically significant strains from the SIG and CoNS groups were selected, along with 1 *S. aureus* strain, based on their intermediate or resistant profiles to at least two antibiotics, as determined by the disk diffusion method (Table A1).

**Figure 3:**
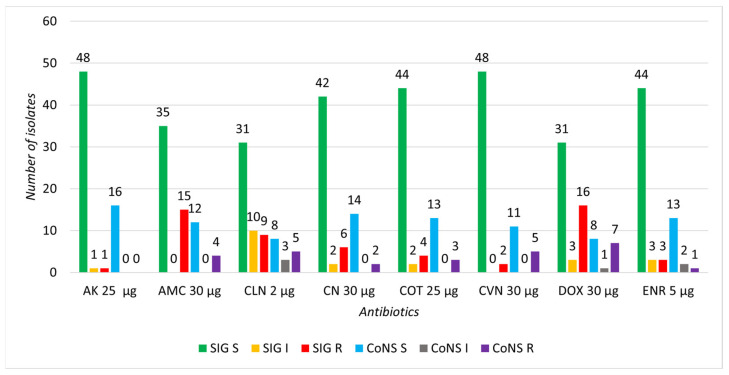
Representation of sensitive, intermediate, and resistant strains of selected SIG and CoNS isolates to individual antibiotics. S—sensitive, I—intermediate, R—resistant.

The highest sensitivity was observed for AK, with 96% of SIG isolates and 100% of CoNS isolates being susceptible. High sensitivity was also noted for CVN in SIG isolates (96%) and CN in CoNS isolates (87.5%). The highest proportion of intermediate isolates was recorded for CLN, with 20% in SIG and 18.75% in CoNS. Regarding resistance, the highest percentage of resistant SIG isolates was observed for DOX (32%) and AMC (30%). In CoNS isolates, the highest resistance was recorded for DOX (43.75%), followed by CVN and CLN (31.25% each) (Figure 3).

Based on the disk diffusion method, we identified four multi-resistant CoNS strains (*S. equorum* (three isolates) and *S. nepalensis* (one isolate)), and four multi-resistant SIG strains, all of which were *S. pseudintermedius*. According to the established criteria, 21 isolates (43.8%; all *S. pseudintermedius*) from SIG were selected for MIC determination, and 10 isolates from CoNS (62.5%; including 2 isolates of *S. felis*, 2 isolates of *S. xylosus*, 6 isolates of *S. equorum*, and 1 isolate of *S. nepalensis*) were also selected for determination. In addition to SIG and CoNS, we also selected *S. aureus* for testing as an important pathogen causing serious skin infections. The *S. aureus* isolate was not resistant to any antibiotic by the disk diffusion method but was intermediate to CLN, CVN, and DOX. It did not show resistance under MIC determination either.

Among the total number of isolates, the highest resistance was observed in ampicillin, erythromycin, clindamycin, chloramphenicol, and tetracycline. The MIC xG (mg/L) for tetracycline was 1.45 mg/L, for clindamycin 0.761 mg/L, for erythromycin 1.429 mg/L, and for ampicillin 0.863 mg/L, which exceeded the EUCAST breakpoints. Oxacillin resistance was observed in 21.2% of isolates, with a MIC xG of 0.240 mg/L (breakpoint 0.25 mg/L).

**Figure 4:**
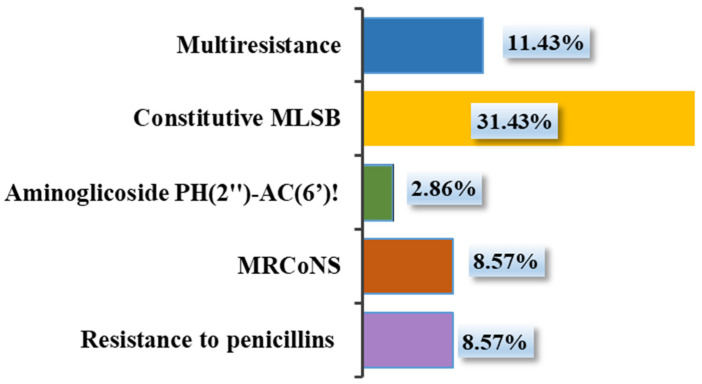
Predicted mechanisms of staphylococcal resistance based on MIC results.

According to the resistance profile, resistance mechanisms were also automatically evaluated. Constitutive MLSB (macrolide/lincosamide/streptogramin B) was the most common (31.43%), but multi-resistant CoNS strains (11.43%) were also present, which represent the highest risk for pet owners. The MRCoNS mechanism (methicillin-resistant CoNS) was recorded in 8.57% of isolates, with all beta-lactams being ineffective. Penicillinase resistance also represented 8.57%. In 2.86% of isolates, a resistance mechanism to aminoglycosides (PH(2″)-AC(6′)!) was found, which represents complications in treatment with these antibiotics and combined enzymatic resistance to gentamicin, tobramycin, and ampicillin (Figure 4).

**Figure 5:**
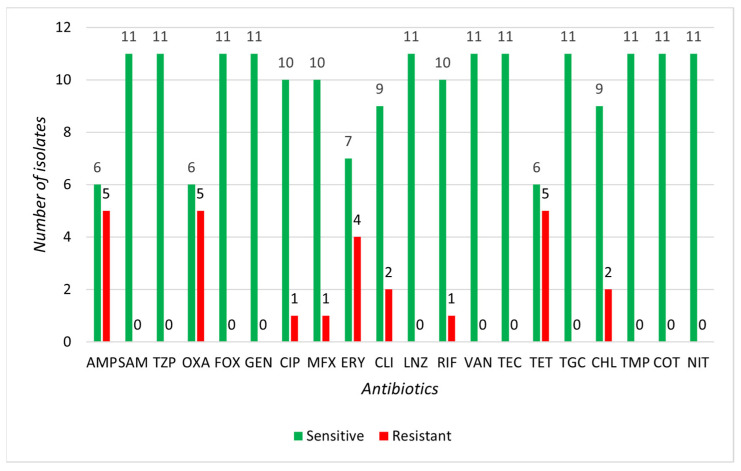
Representation of sensitive and resistant strains of 11 selected CoNS isolates to individual antibiotics.

**Figure 6:**
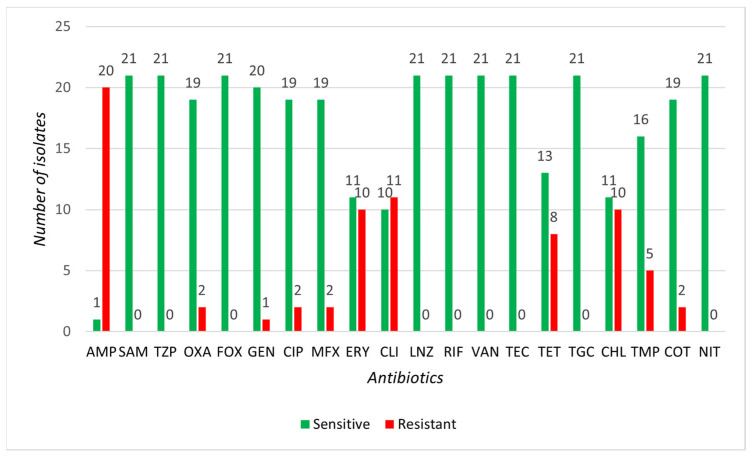
Representation of sensitive and resistant strains of selected 21 SIG isolates to individual antibiotics.

None of the CoNS isolates demonstrated resistance to SAM, TZP, FOX, GEN, LNZ, VAN, TEC, TGC, TMP, COT, or NIT. The highest resistance in CoNS isolates was observed for AMP, OXA, and TET (45.5%), with high resistance also for ERY (36.4%). Resistance to the anti-staphylococcal antibiotic OXA was found in up to five isolates: *S. equorum* (three isolates), *S. xylosus* (one isolate), and *S. nepalensis* (one isolate) (Figure 5). None of the SIG isolates indicated resistance to SAM, TZP, FOX, LNZ, RIF, VAN, TEC, TGC, or NIT. The highest resistance in SIG isolates was observed for AMP (95.2%), with high resistance also noted for CLI (52.4%), ERY, and CHL (47.6% each). Two isolates of *S. pseudintermedius* were resistant to the anti-staphylococcal antibiotic OXA (Figure 6). Based on the MIC method, we identified 4 multi-resistant CoNS strains: *S. equorum* (1 isolate), *S. nepalensis* (1 isolate), and *S. felis* (2 isolates), along with 12 multi-resistant SIG strains, all of which were *S. pseudintermedius* (Table A2).

### 3.3. Gene Resistance Detection of Selected SIG and CoNS Isolates

The *mecA* gene was confirmed in 4 tested isolates (*n* = 33), all of which were representatives of the species *S. equorum*. The *mecC* gene was not detected in any isolate. Neither the *mecA* nor *mecC* genes were detected in the remaining resistant isolates. Isolates that carried the *mecA* gene were phenotypically resistant to penicillins (AMP, AMC, OXA), except isolate no. 75 (Table 1). As control strains, we used *S. aureus* CCM 4750 for *mecA* gene and *S. edaphicus* CCM 8731 for *mecC* gene.

### 3.4. Biofilm Activity Test

The biofilm-forming ability of individual SIG and CoNS isolates was tested using a modified colorimetric method according to O’Toole et al. [37] in 33 clinically relevant isolates (1 *S. aureus*, 11 CoNS, and 21 SIG). Biofilm production was assessed according to Stepanović et al. [38]. Of the 33 isolates tested, 29 (87.9%) were classified as strong biofilm producers, 2 as intermediate biofilm producers (*S. equorum* isolate 45 and *S. pseudintermedius* isolate 105), and 2 as weak biofilm producers (*S. pseudintermedius* isolate 48 and *S. equorum* isolate 75). Among the SIG group, 93.9% of isolates were strong biofilm producers (*S. pseudintermedius*), while, in the CoNS group, 81.8% were strong biofilm producers, including four isolates of *S. equorum*, two isolates of *S. felis*, two isolates of *S. xylosus*, and one isolate of *S. nepalensis*. The isolate confirmed as S. aureus showed strong biofilm activity (Table 2). The positive control was reference strain *S. aureus* CCM 4223, and the negative control was reference strain *S. epidermidis* CCM 4418.

A more detailed analysis of biofilm production, an important virulence factor, was performed at both phenotypic and genotypic levels using multiplex PCR. In SIG and CoNS, no genes associated with biofilm formation (*fnbA*, *fnbB*, *clfA*, *clfB*, and the *icaABCD* operon) were detected. Genes encoding clumping factors (*clfA*, *clfB*) and the *icaABCD* operon (*icaA*, *icaB*, *icaC*) were detected only in *S. aureus*. As a control strain, we used *S. aureus* CCM 4223.

## 4. Discussion

Based on the examination of 108 samples, it was demonstrated that dogs are reservoirs of various *Staphylococcus* spp. (62%), which confirms their commensal occurrence in the skin microbiota, as was also described in the published works where staphylococci were defined as a natural part of the skin microbiota of dogs [1,46,47]. In the samples obtained, staphylococci were isolated mainly in the summer months, when the highest prevalence of both SIG and CoNS was assumed [48,49]. However, these results were very variable depending on the region and the breed of the dog. The available data describes the seasonal occurrence of staphylococci only from human samples. The identified staphylococcal species in the samples were predominant compared with other bacteria, with the highest prevalence (72.1%) recorded by the opportunistic pathogen *S. pseudintermedius* from the SIG group (CoPS), whose occurrence in dogs was the majority compared with other staphylococcal species [11,50]. The highest representation from the CoNS group was *S. equorum*, which comprised 15.2% of all *Staphylococcus* spp. isolates, like the work of the Schmidt et al. [51]. The highest risk in terms of resistance was represented by *S. aureus*, which we identified in one isolate, and, like other authors, its prevalence percentage was below 9%. The reason is that the samples were not collected only from skin pyodermas [52,53]. Due to the different results in identification using biochemical tests (STAPHY test 24) and MALDI-TOF MS, we, also according to the published results of Waneck et al., leaned towards identification based on the evaluation of the protein spectrum using MALDI-TOF MS [54].

With the widespread and irrational use of antibiotics, bacterial infections are once again becoming a threat to public health, humans, animals, and food safety [55]. In connection with the emergence of resistant bacteria, it has become customary to refer to this phenomenon as “an antibiotic resistance crisis” [56]. According to the disk diffusion method, the highest sensitivity of both SIG and CoNS was proven to be to amikacin, in almost all isolates, which was also described by Khinchi et al. [57]. Loeffler et al. and Mack et al. describe sensitivity of staphylococci to cephalosporins up to 97%, which in our isolated SIG was recorded at the level of 96% to cefovecin (third generation) and in CoNS at the level of 87.5% to cephalexin (first generation) [58,59]. In veterinary medicine, a high rate of resistance to amoxicillin (or amoxicillin/clavulanate) and tetracycline antibiotics is observed, which was also confirmed in our study, where the rate of resistance in SIG to amoxicillin/clavulanate was 30%, and to doxycycline 32%, and in CoNS to doxycycline 43.75% [60,61]. In addition, we observed increased resistance in CoNS to cefovecin (third generation) and to clindamycin (31.25%), which correlates with the results of Cunha and Horsman et al. [62,63]. The results also correspond to the consumption of antibiotics in Slovakia and are directly related to the fact that the most frequently prescribed antibiotics in veterinary medicine are beta-lactam antibiotics, tetracyclines, sulfonamides, and pleuromutilins [64]. The prevalence of multi-resistant strains of both SIG (8%) and CoNS (25%) was lower compared with the studies by Lord et al. and Chah et al., but this may be because the samples were collected by the stated objective of monitoring the occurrence of multidrug-resistant staphylococci on the skin of healthy individuals [65,66].

From the isolated staphylococci, in which the disk diffusion method revealed a significant antibiotic resistance profile, MIC was subsequently determined by the microdilution colorimetric method using commercial plates from MIDITECH for 20 types of antibiotics. None of the SIG/CoNS isolates were resistant to the reserve antibiotics used in the MIC determination (linezolid, tigecycline) or to the antibiotics from the “Watch” group (piperacillin/tazobactam, cefoxitin, vancomycin, teicoplanin), classified according to the WHO AWaRe classification of antibiotics for evaluation and monitoring of use document from 2023 [67]. Similarly, no resistance was demonstrated in SIG or CoNS to ampicillin/sulbactam, cefoxitin, or nitrofurantoin. The highest resistance rate in SIG (95.2%) and CoNS (45.5%) was observed with ampicillin without a beta-lactamase inhibitor, which corresponds to the high resistance rate to these antibiotics and is directly related to the high consumption of penicillins (especially aminopenicillins) in veterinary medicine [65]. In addition to resistance to penicillins, SIG also showed high resistance rates to antibiotics from the lincosamide group (clindamycin—52.4%), macrolides (erythromycin—47.6%), and amphenicols (chloramphenicol—47.6%). The studies by Gronthal et al. and Ganiere et al. reported lower resistance rates to these antibiotics, where a lower prevalence of resistant strains was assumed, which may not correspond to the current situation [68,69]. Currently, only a limited amount of resistance data is available in SIG. One of the more recent studies is the work published by Humphrey et al., where the authors report similar results, except for clindamycin, for which they report a higher percentage of resistance than we observed [70]. Only 9.5% of SIG isolates were resistant to the anti-staphylococcal antibiotic oxacillin, which roughly correlates with the results of Grönthal et al., reporting 14% resistance [68]. Of the 21 isolates obtained, 12 (57.1%) were defined as multidrug-resistant (MDR) strains of *S. pseudintermedius*. Available studies focus not only on the occurrence in companion animals but also on the occurrence and mutual comparison of their owners since these are potentially zoonotic bacteria [71]. In CoNS, in comparison with SIG, in addition to resistance to erythromycin (36.4%), a high rate of resistance to oxacillin (45.5%) and tetracycline (45.5%) was observed. Resistance of CoNS and SIG to these antibiotics is poorly described, and the prevalence of resistant strains is variable. If it is recorded, it is only at a low level, and studies, like those for SIG, are performed on dogs and their owners [72,73]. The prevalence of MDR CoNS strains obtained by us (36%) is comparable to the study by Schmidt et al., and the isolated MDR strains were within the species *S. equorum*, *S. nepalensis*, and *S. felis* [51]. The results obtained in this study indicate the prevalence of resistant staphylococci mainly from the SIG group to antibiotics (ampicillin, erythromycin, clindamycin, chloramphenicol, and tetracycline), which are widely used in veterinary and human practice in the Central European region [64].

The prevalence of methicillin-resistant CoNS in dogs has not been described but has been described in older studies in chickens, cattle, and humans. The prevalence was only around 16% [74,75,76]. Up to 75% resistance to the anti-staphylococcal antibiotic oxacillin was observed in our CoNS isolates. The *mecA* resistance gene was only determined in *S. equorum*, a CoNS strain, which has not yet been described in the available literature. The observed increased resistance to oxacillin was probably caused by a different mechanism. As reported by Platenik et al., *mecC* was not detected in our study either due to its extremely low prevalence [77,78,79]. One amoxicillin/clavulanate-resistant isolate carried the *mecA* gene, which encodes PBP 2a with lower affinity for beta-lactam antibiotics, which means that the concentration of the antibiotic penetrating the cell is reduced, due to which the inhibitory effect of clavulanic acid is insufficient. The zoonotic potential of staphylococci and the range of the community and hospital spread make them one of the most important nosocomial pathogens [5,80,81]. These species, especially their resistant strains, can cause serious infections in immunocompromised hosts [5].

Although the main virulence factors responsible for staphylococcal pathogenicity were initially identified and characterized in S. aureus, recently published results indicate their presence in many bacterial species of other CoPS and CoNS [7,82]. One important virulence factor is biofilm production [83]. Bacterial cells of staphylococci are arranged in a multilayered tower-like biofilm, in which antibiotic resistance is due not only to poor diffusion of drugs but also to reduced metabolic activity of persistent cells in the lower layers of the biofilm [20,21]. Jantorn et al. observed strong and moderate biofilm production in 90.55% and Wang et al. in 89.66% of SIG (*S. pseudintermedius*) isolates [82,84]. Our results showed similar strong and moderate biofilm production in 96.9% of SIG isolates. We also observed high biofilm formation ability in CoNS, namely in 93.8% of isolates (only one isolate was a weak biofilm producer); in Silva et al., all CoNS produced biofilm without determining the rate of its production [85]. Biofilm production in the *S. aureus* isolate was determined phenotypically, and genes involved in the biofilm formation process (*clfA*, *clfB*, *icaA*, *icaB*, *icaC*) were detected at the genotypic level. However, these genes were not observed in strong and moderate biofilm producers in other *Staphylococcus* spp., indicating that the biofilm formation process may not depend on the products of these genes [86].

## 5. Conclusions

This study contributed to a global view of SIG and CoNS as bacteria commonly colonizing the dog’s skin. Staphylococci occurring on the skin of dogs show resistance to commonly used antibiotics, which may pose a risk not only to the shelter but also to shelter employees and potential adoptive parents, especially when it comes to immunocompromised individuals. The risk of the findings lies mainly in the results, where we clarified that, on the skin of shelter dogs without any clinical symptoms, there are also multi-resistant strains of staphylococci, which are carriers of resistance genes with a high ability to produce biofilm, which also contributes to an increased level of antibiotic resistance. With the results, we contribute to the description of the current state and point out the risk of the emergence and risk of transmission of strains that are thus able to transfer genes of virulence and resistance factors by horizontal gene transfer between species. Moreover, after mutual colonization with other bacterial species, they may acquire additional virulence factors and new atypical resistance mechanisms. The results obtained in this study not only fill a gap in the current literature but also help to understand the dynamics of bacterial infections in veterinary medicine and lay the foundation for future studies investigating this issue.

## Figures and Tables

**Table 1:** Susceptibility of isolates to selected types of antibiotics by the Kirby–Bauer method.

Isolate with *mecA* Gene	MHA	MIC
*S. equorum* no. 43	CLN, CVN, DOX	AMP, OXA, ERY, TET
*S. equorum* no. 45	CLN, CVN	ERY, CLN
*S. equorum* no. 50	AMC, CLN	AMP, OXA
*S. equorum* no. 75	CLN, CN, COT, CVN, DOX	-

**Table 2:** Evaluation of biofilm formation in *Staphylococcus* spp. isolates.

**CoNS**	**Isolate**	**Diameter**	**Biofilm**
	2	3.657	strong
	23	3.620	strong
	30	3.637	strong
	35	3.661	strong
	43	2.605	strong
	44	2.55	strong
	45	1.563	moderate
	50	3.117	strong
	60	3.543	strong
	75	0.873	weak
	93	2.731	strong
**SIG**	4	3.687	strong
	6	3.525	strong
	11	3.637	strong
	16	3.722	strong
	24	3.536	strong
	33	3.677	strong
	36	3.608	strong
	42	3.435	strong
	46	3.528	strong
	48	0.566	weak
	52	3.629	strong
	56	3.574	strong
	57	3.561	strong
	58	3.300	strong
	65	3.495	strong
	80	3.304	strong
	82	2.143	strong
	85	3.557	strong
	88	3.670	strong
	99	3.525	strong
	105	1.303	moderate
**SA**	71	2.444	strong

Negative control ODc = 0.441022, non-biofilm forming strain (OD ≤ ODc), weak biofilm forming strain (ODc < OD ≤ 2 × ODc), moderate biofilm-forming strain (2 × ODc < OD ≤ 4 × ODc), strong biofilm forming strain (4 × ODc < OD) [38]. SA—*S. aureus*.

## Data Availability

The original contributions presented in this study are included in the article. Further inquiries can be directed to the corresponding author.

## Abbreviations

The following abbreviations are used in this manuscript:

AIP	auto-inducting peptide
AK	amikacin
AMC	amoxicillin/clavulanate
AMP	ampicillin
BPA	Baird-Parker agar
CIP	ciprofloxacin
CLI, CLN	clindamycin
CN	cephalexin
CoNS	coagulase-negative staphylococci
CoPS	coagulase-positive staphylococci
COT	co-trimoxazole
CVN	cefovecin
CWAPs	cell wall-anchored proteins
DNA	deoxyribonucleic acid
DOX	doxycycline
EPS	extracellular polymeric substance
ENR	enrofloxacin
ERY	erythromycin
ESBL	extended-spectrum beta-lactamase
EUCAST	European Committee on Antimicrobial Susceptibility Testing
FOX	cefoxitin
GEN	gentamicin
CHL	chloramphenicol
LNZ	linezolid
MALDI-TOF MS	matrix-assisted laser desorption ionization–time of flight mass spectrometry
MDR	multidrug-resistant
mBHI	modified brain heart infusion
MFX	moxifloxacin
MGE	mobile genetic element
MHA	Mueller–Hinton agar
MIC	minimum inhibitory concentration
MRCoNS	Multi-resistant CoNS
MRSA	methicillin-resistant *Staphylococcus aureus*
MSA	mannitol salt agar
MSCRAMM	microbial surface components recognizing
NIT	nitrofurantoin
OD	optical density
OXA	oxacillin
PBP	penicillin-binding protein
PCR	polymerase chain reaction
PIA	polysaccharide intercellular adhesin
QS	quorum sensing
RIF	rifampicin
rRNA	ribosomal ribonucleic acid
SAM	ampicillin/sulbactam
*SCCmec*	staphylococcal cassette chromosome *mec*
SIG	*Staphylococcus intermedius* group
TEC	teicoplanin
TGC	tigecycline
TET	tetracycline
TMP	trimethoprim
TZP	piperacillin/tazobactam
VAN	vancomycin
VRSA	vancomycin-resistant *Staphylococcus aureus*

## Appendix A

**Table A1:** Susceptibility of isolates to selected types of antibiotics by the Kirby–Bauer method.

	Species	AK 25 µg	AMC 30 µg	CLN 2 µg	CN 30 µg	COT 25 µg	CVN 30 µg	DOX 30 µg	ENR 5 µg
**CoNS**									
2	*S. felis* *	S	S	I	S	S	S	R	S
23	*S. xylosus* *	S	R	S	S	S	S	R	S
30	*S. felis* *	S	R	S	S	R	S	S	S
32	*S. simulans*	S	S	S	S	S	S	S	S
35	*S. xylosus* *	S	R	R	S	S	S	S	S
39	*S. equorum*	S	S	S	S	S	S	R	S
41	*S. equorum*	S	S	S	S	S	R	S	S
43	*S. equorum* *	S	S	R	S	S	R	R	S
44	*S. equorum* *	S	S	S	S	S	S	I	I
45	*S. equorum* *	S	S	R	S	S	R	S	I
50	*S. equorum* *	S	R	R	S	S	S	S	S
60	*S. equorum* *	S	S	I	R	S	R	R	S
75	*S. equorum* *	S	S	R	R	R	R	R	S
76	*S. equorum*	S	S	S	S	S	S	S	S
91	*S. nepalensis*	S	S	S	S	S	S	S	S
93	*S. nepalensis* *	S	S	I	S	R	S	R	R
**SIG**									
3	*S. pseudintermedius*	S	S	S	S	S	S	S	S
4	*S. pseudintermedius* *	S	S	I	S	S	S	R	R
5	*S. pseudintermedius*	S	S	I	S	S	S	S	S
6	*S. pseudintermedius* *	S	S	R	S	S	S	R	S
7	*S. pseudintermedius*	S	S	S	S	S	S	I	S
9	*S. pseudintermedius*	S	S	S	R	S	S	S	I
10	*S. pseudintermedius*	S	S	I	S	S	S	S	S
11	*S. pseudintermedius* *	S	R	S	S	S	S	R	S
13	*S. pseudintermedius*	S	R	S	S	S	S	S	S
14	*S. pseudintermedius*	S	R	S	S	S	S	S	S
16	*S. pseudintermedius* *	S	R	I	S	S	S	R	R
17	*S. delphini*	S	S	S	S	S	S	R	S
18	*S. pseudintermedius*	S	S	S	S	S	S	S	S
19	*S. pseudintermedius*	S	S	S	S	S	S	S	S
20	*S. pseudintermedius*	S	S	S	S	S	S	S	S
21	*S. pseudintermedius*	S	R	S	S	S	S	S	S
22	*S. pseudintermedius*	S	S	S	S	S	S	S	S
24	*S. pseudintermedius* *	S	R	S	S	S	S	R	S
25	*S. pseudintermedius*	S	S	S	S	S	S	R	S
27	*S. pseudintermedius*	S	S	S	S	S	S	S	S
31	*S. pseudintermedius*	S	S	R	S	S	S	S	S
33	*S. pseudintermedius* *	S	S	R	S	R	S	S	S
36	*S. pseudintermedius* *	S	S	S	R	S	S	S	I
37	*S. pseudintermedius*	S	R	S	S	S	S	S	S
42	*S. pseudintermedius* *	S	R	R	S	S	S	R	S
46	*S. pseudintermedius* *	S	S	I	I	S	R	I	S
48	*S. pseudintermedius* *	R	R	R	R	S	R	R	S
52	*S. pseudintermedius* *	S	R	R	S	S	S	R	S
53	*S. pseudintermedius*	S	S	S	S	R	S	S	S
55	*S. pseudintermedius*	S	S	S	S	S	S	S	S
56	*S. pseudintermedius* *	S	R	R	S	S	S	S	S
57	*S. pseudintermedius* *	S	S	I	R	I	S	S	R
58	*S. pseudintermedius* *	S	R	R	S	S	S	S	S
65	*S. pseudintermedius* *	S	R	S	S	S	S	R	S
66	*S. pseudintermedius*	S	S	R	S	S	S	S	S
67	*S. pseudintermedius*	I	S	S	S	S	S	S	S
73	*S. pseudintermedius*	S	S	I	S	S	S	S	S
80	*S. pseudintermedius* *	S	R	I	S	S	S	R	S
81	*S. pseudintermedius*	S	S	S	S	S	S	I	S
82	*S. pseudintermedius* *	S	S	S	S	R	S	R	S
85	*S. pseudintermedius* *	S	S	I	S	R	S	R	I
88	*S. pseudintermedius* *	S	S	S	R	S	S	R	S
90	*S. pseudintermedius*	S	S	S	R	S	S	S	S
92	*S. pseudintermedius*	S	S	S	S	S	S	S	S
96	*S. pseudintermedius*	S	S	I	S	S	S	S	S
99	*S. pseudintermedius* *	S	R	S	S	I	S	S	S
100	*S. pseudintermedius*	S	S	S	S	S	S	R	S
103	*S. pseudintermedius*	S	S	R	S	S	S	S	S
104	*S. pseudintermedius*	S	S	S	I	S	S	S	S
105	*S. pseudintermedius* *	S	S	S	S	S	S	S	S
**Other CoPS**									
71	*S. aureus**	S	S	I	S	S	I	I	S

S—sensitive, I—intermediate, R—resistant; * = clinically significant isolate.

**Table A2:** MIC profile of selected *Staphylococcus* spp. isolates.

	AMP	SAM	TZP	OXA	FOX	GEN	CIP	MFX	ERY	CLI	LNZ	RIF	VAN	TEC	TET	TGC	CHL	TMP	COT	NIT
**CoNS**																				
2 *	R	S	S	S	S	S	S	S	S	S	S	R	S	S	R	S	S	S	S	S
23	S	S	S	S	S	S	S	S	S	S	S	S	S	S	S	S	S	S	S	S
30 *	R	S	S	S	S	S	S	S	R	R	S	S	S	S	R	S	R	S	S	S
35	R	S	S	R	S	S	S	S	S	S	S	S	S	S	S	S	S	S	S	S
43 *	R	S	S	R	S	S	S	S	R	S	S	S	S	S	R	S	S	S	S	S
44	S	S	S	R	S	S	S	S	S	S	S	S	S	S	S	S	S	S	S	S
45	S	S	S	S	S	S	S	S	R	R	S	S	S	S	S	S	S	S	S	S
50	R	S	S	R	S	S	S	S	S	S	S	S	S	S	S	S	S	S	S	S
60	S	S	S	S	S	S	S	S	S	S	S	S	S	S	R	S	S	S	S	S
75	S	S	S	S	S	S	S	S	S	S	S	S	S	S	S	S	S	S	S	S
93 *	S	S	S	R	S	S	R	R	R	S	S	S	S	S	R	S	R	S	S	S
**SIG**																				
4 *	R	S	S	S	S	S	R	R	R	R	S	S	S	S	R	S	R	S	S	S
6 *	R	S	S	S	S	S	S	S	S	S	S	S	S	S	S	S	S	R	R	S
11	R	S	S	S	S	S	S	S	S	S	S	S	S	S	R	S	S	S	S	S
16 *	R	S	S	S	S	S	R	R	R	R	S	S	S	S	R	S	R	S	S	S
24 *	R	S	S	S	S	S	S	S	R	R	S	S	S	S	S	S	R	S	S	S
33 *	R	S	S	R	S	S	S	S	R	R	S	S	S	S	S	S	R	S	S	S
36	R	S	S	S	S	S	S	S	S	S	S	S	S	S	R	S	S	S	S	S
42 *	R	S	S	S	S	S	S	S	R	R	S	S	S	S	R	S	R	R	R	S
46 *	R	S	S	S	S	S	S	S	R	R	S	S	S	S	S	S	R	S	S	S
48	R	S	S	S	S	S	S	S	S	S	S	S	S	S	R	S	S	S	S	S
52 *	R	S	S	S	S	S	S	S	R	R	S	S	S	S	S	S	R	S	S	S
56 *	R	S	S	R	S	S	S	S	R	R	S	S	S	S	S	S	R	R	S	S
57 *	R	S	S	S	S	S	S	S	R	R	S	S	S	S	S	S	R	S	S	S
58	R	S	S	S	S	S	S	S	S	R	S	S	S	S	S	S	S	S	S	S
65	S	S	S	S	S	S	S	S	S	S	S	S	S	S	R	S	S	S	S	S
80	R	S	S	S	S	S	S	S	S	S	S	S	S	S	S	S	S	S	S	S
82 *	R	S	S	S	S	R	S	S	S	S	S	S	S	S	S	S	S	R	S	S
85	R	S	S	S	S	S	S	S	S	S	S	S	S	S	R	S	S	S	S	S
88	R	S	S	S	S	S	S	S	S	S	S	S	S	S	S	S	S	R	S	S
99 *	R	S	S	S	S	S	S	S	R	R	S	S	S	S	S	S	R	S	S	S
105	R	S	S	S	S	S	S	S	S	S	S	S	S	S	S	S	S	S	S	S
***S. aureus***																				
71	S	S	S	S	S	S	S	S	S	S	S	S	S	S	S	S	S	S	S	S

* = multi-resistant strain; R = resistant strain; S = sensitive strain.
